# Elevated blood malondialdehyde level contributed to a high stroke risk in a Chinese elderly population from rural areas: a cross-sectional study

**DOI:** 10.1038/s41598-024-54419-9

**Published:** 2024-02-21

**Authors:** Rong Wan, Yuhao Su, Meilan Zhu, Ying Huang

**Affiliations:** 1 Rehabilitation Department, The Second Affiliated Hospital, Jiangxi Medical College, Nanchang University, Nanchang, 330006 Jiangxi China; 2 Cardiovascular Department, The Second Affiliated Hospital, Jiangxi Medical College, Nanchang University, Nanchang, 330006 Jiangxi China; 3 Jiangxi Key Laboratory of Molecular Medicine, The Second Affiliated Hospital, Jiangxi Medical College, Nanchang University, Nanchang, 330006 Jiangxi China

**Keywords:** Malondialdehyde, Oxidative damage, Stroke, Reactive oxygen species, Older adults, Biochemistry, Biomarkers

## Abstract

Individuals living in rural areas have a higher incidence rate of stroke than their urban counterparts in China. However, few studies have investigated the association between blood malondialdehyde (MDA), an end product of lipid oxidation caused by reactive oxygen species (ROS), and stroke risk in rural populations. We aimed to investigate whether blood MDA levels contribute to a higher stroke risk in a Chinese elderly population from rural areas. Data from 2011 to 2012 from the Chinese Longitudinal Healthy Longevity Survey (CLHLS), a national cohort of older adults in China, were analyzed. Smooth curve and multivariable correction analyses were used to evaluate the association between blood MDA levels and stroke risk in elderly populations from rural and urban areas, respectively. The median age of all included participants (N = 1598) was 84.04 years. The results of the smooth curve model revealed a gradual upward trend in the association of blood MDA levels with stroke risk in rural participants but not in urban participants. Similarly, the conditional logistic regression analysis suggested a significant association between MDA levels and stroke risk in rural participants but not in urban participants after adjustments for related confounding factors (age, sex, current smoker, current drinker, regular exercise, BMI and cardiovascular diseases (hypertension, heart disease, atrial fibrillation and diabetes)) were made. In brief, among the elderly population in China, elevated blood MDA levels were associated with increased stroke risk in rural participants but not in urban participants.

## Introduction

The urban‒rural disparity in life expectancy has gradually expanded in recent decades, especially in developing countries^1,2^. An important factor that widens the gap is an imbalance in the morbidity and mortality of cardiovascular diseases (CVDs), particularly stroke, in urban and rural areas^3^. A prior study reported that 7.1% of a gap in urban‒rural disparities in life expectancy resulted from stroke, and rural areas had a significantly higher rate of stroke mortality than urban areas^4^. However, the mechanisms underlying urban‒rural differences in the incidence rate of stroke are incompletely known. Some previous data might partly explain the difference. For example, individuals who lived in rural areas tended to have more and worse risk factor profiles, including hypertension, diabetes and dyslipidemia for stroke, compared with those who lived in urban areas^5–9^. Other evidence also suggested that populations in rural areas had less access to medical services than populations in urban areas, which may lead to an increase in the incidence rate of stroke^10,11^. Much of this literature, however, has mainly focused on the relationship between risk factors for urban‒rural differences and stroke, such as demographic characteristics and risk factors related to lifestyle. Few studies have investigated the association of biochemical indices in the human body with stroke risk in rural and urban populations.

Oxidative damage can arise from an imbalance between increased production of reactive oxygen species (ROS) and/or a reduction in antioxidant defenses^12,13^. Malondialdehyde (MDA), the main end product of lipid oxidation, can cause cross-linking polymerization of proteins, nucleic acids and other living macromolecules and cause great damage to the activities of the mitochondrial respiratory chain complex and key enzymes in mitochondria^14,15^. The present evidence has demonstrated that rapid ROS overexpression can immediately overwhelm antioxidant defenses after acute ischemic stroke, further causing tissue or cell damage. Furthermore, a second burst of ROS generation caused by increased blood flow contributes to reperfusion injury in brain tissue^16^. As an important indicator of the degree of oxidative damage, MDA has also been found to be associated with a series of complications after stroke^17,18^. However, it is not yet known whether there is an urban‒rural difference in the association between blood MDA and stroke risk.

Considering the present research background, we aimed to determine the association between blood MDA levels and stroke risk in a Chinese elderly population from the Chinese Longitudinal Healthy Longevity Survey (CLHLS). Our first aim was to investigate whether a higher blood MDA level contributed to elevated stroke risk in the elderly population, independent of confounding factors such as demographic characteristics, lifestyle risks and concomitant CVDs. Then, we further investigated whether there was an urban‒rural disparity in the relationship between blood MDA levels and stroke in the present study.

## Methods

### Study population

Study data were from the CLHLS study (https://www.icpsr.umich.edu/web/pages/ICPSR/), which is a prospective, longitudinal, community-based study^19^. Twenty-two provinces in China were initially selected, and then half of the cities or counties in these provinces were randomly selected to include the study population. A detailed description of the CLHLS has been published elsewhere^20^. Further details on the study design, study procedures and data quality assessment were also performed elsewhere^21–23^. In the present study, we used data from the 2011–2012 wave of the cohort. Our study had a sufficient analysis among adults aged 65 and older in the wave with complete information on stroke, MDA and other covariates. In all, 9765 elderly individuals were initially included in the 2011–2012 CLHLS. For the purpose of this study, 1598 elderly participants met the inclusion criteria in our study after excluding incorrect and missing data. All participants or their relatives were informed of the data for research in the CLHLS study. Written informed consent was obtained from all participants or their proxies, according to the Declaration of Helsinki guidelines.

### Assessment of covariates

A structured questionnaire was conducted to obtain covariates. Sociodemographic characteristics included age, gender and residence. The other health characteristics included smoking, drinking, activities of daily living (regular exercise), body mass index (BMI) and self-reported diseases, such as heart disease, stroke, diabetes mellitus and other CVDs. Residence was categorized as “urban (city residence or town)” or “rural (countryside residence)”. Current smokers and current drinkers were evaluated by the self-reports “Do you currently smoker?” and “Do you currently drink alcohol?” Current smokers were categorized as “current smokers” and “not current smokers”. Current drinker was categorized as “current drinker” and “not current drinker”. Activity of daily living was classified as “regular exercise” and “not regular exercise”, according to “Do you do exercise regularly at present (yes or not)?” Self-reported diseases were classified as “yes” and “not”. The definition of all suffered diseases has been clarified in previous studies^19–23^.

### Statistical analysis

All of the analyses were performed by using EmpowerStats 3.0. A *P* value ≤ 0.05 was considered to be statistically significant. We first implemented a smooth curve to estimate the association between blood MDA levels and stroke risk. Then, stratification analysis using a smooth curve was further performed to evaluate the association between blood MDA levels and the risk of stroke in the elderly population from rural and urban areas. Furthermore, in the stratification analysis, multiple logistic regression analysis was used to analyze the association of blood MDA with stroke risk in rural and urban areas respectively. From the Crude model to Model 4, variables including age, gender, current smoker, current drinker, regular exercise, BMI and CVDs (hypertension, heart disease, atrial fibrillation and diabetes) were controlled. Crude: Not adjusted. Model 1: Adjusted for age and gender; Model 2: Adjusted for age, gender, current smoker, current drinker and regular exercise; Model 3: Adjusted for age, gender, current smoker, current drinker, regular exercise and BMI; Model 4: Adjusted for age, gender, current smoker, current drinker, regular exercise, BMI and CVDs.

Additionally, stratification analysis for the association between blood MDA level and stroke risk was also performed by using “age”, “gender”, “current smoker”, “current drinker”, “regular exercise” and “BMI” as the hierarchical variables.

### Ethics approval and consent to participate

Our study methods were also carried out in accordance with relevant guidelines and regulations. Our data were freely obtained from 2011 to 2012 of the CLHLS study, a national cohort of older adults in China. All participants or their relatives were informed of the data for research in the CLHLS study. Written informed consent was obtained from all participants or their proxies, according to the Declaration of Helsinki guidelines.

## Results

### Characteristics of participants

As shown in Table 1, the mean age of the participants was 84.04 years. A total of 818 of them (51.19%) were male. The mean MDA levels of both urban and rural participants were 4.73 mmol/mL and 5.25 mmol/mL, respectively. All participants were divided into two groups according to urban and rural areas. Compared with urban participants [stroke rate for 16 (6.69%)], rural participants [stroke rate for 105 (7.73%)] were more likely to be current drinkers and tended to have significantly higher serum MDA levels, a higher rate of atrial fibrillation and a lower heart rate. The blood biomarkers are also described in detail in Table 1.

**Table 1:** Characteristics of participants (n = 1598).

Variables	All N = 1598	City and town N = 239	Rural N = 1359	P value
Age (years)	84.04 ± 12.85	82.80 ± 12.24	84.26 ± 12.94	0.101
Gender, Male, n (%)	818 (51.19%)	122 (51.05%)	696 (51.21%)	0.962
BMI (kg/cm^2^)	21.98 ± 13.38	22.11 ± 9.43	21.95 ± 13.96	0.527
BMI < 25, normal, n (%)	1355 (84.79%)	202 (84.52%)	1153 (84.84%)	0.898
BMI ≥ 25, overweight, n (%)	243 (15.21%)	37 (15.48%)	206 (15.16%)	
Heart rate	74.39 ± 12.46	75.92 ± 12.61	74.12 ± 12.42	0.013
Current smoker, n (%)	288 (18.02%)	37 (15.48%)	251 (18.47%)	0.268
Current drinker, n (%)	291 (18.21%)	29 (12.13%)	262 (19.28%)	0.008
Regular exercise, n (%)	282 (17.65%)	45 (18.83%)	237 (17.44%)	0.603
Stroke, n (%)	121 (7.57%)	16 (6.69%)	105 (7.73%)	0.578
Suffering from other CVDs				
Atrial fibrillation, n (%)	151 (9.45%)	14 (5.86%)	137 (10.08%)	0.040
Hypertension, n (%)	434 (27.16%)	74 (30.96%)	360 (26.49%)	0.152
Heart disease, n (%)	127 (7.95%)	17 (7.11%)	110 (8.09%)	0.605
Diabetes, n (%)	42 (2.63%)	8 (3.35%)	34 (2.50%)	0.451
Blood biomarker				
White cell count (10^9^/L)	5.56 ± 1.74	5.73 ± 1.84	5.53 ± 1.72	0.076
Red cell count (10^12^/L)	4.96 ± 17.43	4.29 ± 0.92	5.08 ± 18.90	< 0.001
Hemoglobin (g/L)	126.26 ± 22.82	124.37 ± 23.13	126.59 ± 22.75	0.090
Albumin (g/L)	40.60 ± 4.89	41.59 ± 4.85	40.42 ± 4.88	< 0.001
hsCRP (mg/L)	3.18 ± 8.07	3.88 ± 9.11	3.05 ± 7.87	< 0.001
Creatine (mmol/L)	81.72 ± 28.68	89.36 ± 31.89	80.38 ± 27.88	< 0.001
Total cholesterol (mmol/L)	4.31 ± 0.98	4.29 ± 0.86	4.32 ± 1.00	0.991
HDLC (mmol/L)	1.31 ± 0.36	1.31 ± 0.39	1.31 ± 0.36	0.804
LDLC (mmol/L)	2.55 ± 0.81	2.43 ± 0.76	2.58 ± 0.82	0.021
Glucose (mmol/L)	4.70 ± 2.12	4.40 ± 2.00	4.75 ± 2.13	0.008
MDA (mmol/ml)	5.17 ± 2.06	4.73 ± 1.74	5.25 ± 2.11	< 0.001

BMI, body mass index; CVDs, cardiovascular diseases; CRP, C-reactive protein; LDLC, low-density lipoprotein cholesterol; HDLC, high-density lipoprotein cholesterol; MDA, malondialdehyde.

### Smooth curve analysis of the association between blood MDA level and stroke risk

**Figure 1:**
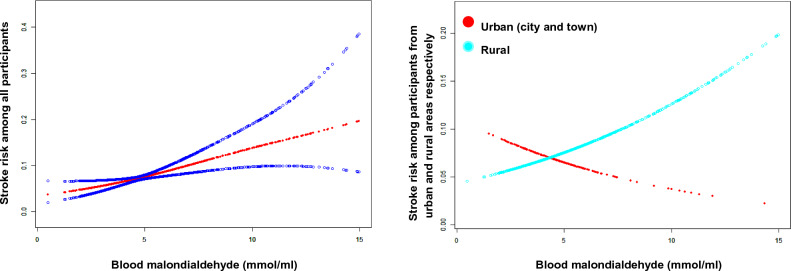
(**A**) The association between blood malondialdehyde (MDA) levels and risk of stroke in all included participants (urban and rural areas). (**B)** The associations between blood malondialdehyde (MDA) levels and risk of stroke in participants from urban and rural areas.

The results analyzed by a smooth curve revealed a gradual upward trend in the association between serum MDA levels and the risk of stroke (Fig. 1A, P < 0.05), which suggested that elevated levels of serum MDA might be closely related to a higher risk of stroke. Furthermore, a gradual upward trend in the association was observed only in rural participants (P < 0.05) and not in urban participants (P > 0.05), as shown in Fig. 1B.

### Elevated blood MDA levels contributed to higher stroke risk in rural participants

Consistent with the above, similar results were analyzed in the multiple logistic regression analysis: elevated blood MDA levels were associated with an increased risk of stroke in Model 4 (OR = 1.13, 95% CI 1.05–1.22, P = 0.002) after adjustments for age, gender, current smoker, current drinker, regular exercise, BMI and CVDs (hypertension, heart disease, atrial fibrillation and diabetes) were made (Table 2). Then, we further investigated whether there was an urban‒rural disparity in the association. Conditional logistic regression analysis suggested that after adjustment for confounding factors, there was a significant association between MDA level and stroke risk only in rural participants (OR = 1.14, 95% CI 1.05–1.24, P = 0.001, Model 4) but not in urban participants (OR = 0.94, 95% CI 0.68–1.29, P = 0.694, Model 4), as shown in Table 3. These results demonstrated an urban‒rural difference in the association between blood MDA levels and stroke in the Chinese elderly population.

**Table 2:** Multiple logistic regression analysis for relationship between blood MDA level and stroke risk in all included participants (N = 1598).

Variables	OR, 95%CI	PerSD change	P value
Crude	1.15 (1.07–1.24)	1.34 (1.15–1.56)	< 0.001
Model 1	1.15 (1.07–1.24)	1.34 (1.15–1.57)	< 0.001
Model 2	1.14 (1.06–1.23)	1.32 (1.13–1.54)	< 0.001
Model 3	1.14 (1.06–1.23)	1.32 (1.13–1.54)	< 0.001
Model 4	1.13 (1.05–1.22)	1.28 (1.10–1.50)	0.002

Crude: Not adjusted.

Model 1: Adjusted for age and gender.

Model 2: Adjusted for age, gender, current smoker, current drinker and regular exercise.

Model 3: Adjusted for age, gender, current smoker, current drinker, regular exercise and BMI.

Model 4: Adjusted for age, gender, current smoker, current drinker, regular exercise, BMI and CVDs (hypertension, heart disease, atrial fibrillation and diabetes).

MDA, malondialdehyde; BMI, body mass index; CVDs, cardiovascular diseases.

**Table 3:** Multiple logistic regression analysis for relationship between blood MDA level and stroke risk in participants rural areas and city & town respectively.

Variables	OR-95%CI	PerSD change	P value
Rural areas			
Crude	1.17 (1.08–1.27)	1.40 (1.19–1.65)	< 0.001
Model 1	1.17 (1.08–1.27)	1.40 (1.19–1.65)	< 0.001
Model 2	1.16 (1.07–1.26)	1.37 (1.16–1.62)	< 0.001
Model 3	1.16 (1.07–1.26)	1.37 (1.16–1.62)	< 0.001
Model 4	1.14 (1.05–1.24)	1.33 (1.12–1.57)	0.001
City& town			
Crude	0.90 (0.65–1.26)	0.84 (0.47–1.50)	0.557
Model 1	0.91 (0.65–1.27)	0.85 (0.48–1.51)	0.575
Model 2	0.90 (0.64–1.25)	0.83 (0.46–1.48)	0.523
Model 3	0.90 (0.64–1.25)	0.83 (0.46–1.48)	0.525
Model 4	0.94 (0.68–1.29)	0.89 (0.51–1.56)	0.694

Crude: Not adjusted.

Model 1: Adjusted for age and gender.

Model 2: Adjusted for age, gender, current smoker, current drinker and regular exercise.

Model 3: Adjusted for age, gender, current smoker, current drinker, regular exercise and BMI.

Model 4: Adjusted for age, gender, current smoker, current drinker, regular exercise, BMI and CVDs (hypertension, heart disease, atrial fibrillation and diabetes).

MDA, malondialdehyde; BMI, body mass index; CVDs, cardiovascular diseases.

Additionally, the association between blood MDA level and stroke risk was further performed by stratified analysis using “age”, “gender”, “current smoker”, “current drinker”, “regular exercise” and “BMI” as the hierarchical variables. Our results showed that age, current drinker, regular exercise and BMI had significant effects on the association between blood MDA levels and stroke risk in rural participants (Table 4). However, in participants from City & Town, we did not observe that any variable had a significant effect on the association between MDA level and stroke risk (Table 5).

**Table 4:** Stratified analysis for relationship between blood MDA level and stroke risk in participants from rural areas (N = 1359).

Variables	N	OR, 95%CI	PerSD change	P value
Age				
Age < 84	655	1.20 (1.08, 1.34)	1.48 (1.18, 1.86)	< 0.001
Age ≥ 84	704	1.09 (0.96, 1.24)	1.20 (0.91, 1.58)	0.186
Gender				
Male	663	1.13 (1.02, 1.26)	1.30 (1.04, 1.64)	0.024
Female	696	1.15 (1.02, 1.31)	1.35 (1.04, 1.75)	0.022
Current smoker				
Yes	251	1.37 (1.08, 1.73)	1.93 (1.17, 3.17)	0.010
No	1108	1.12 (1.02, 1.22)	1.27 (1.05, 1.53)	0.013
Current drinker				
Yes	262	1.27 (0.94, 1.71)	1.65 (0.87, 3.11)	0.126
No	1097	1.14 (1.04, 1.24)	1.31 (1.09, 1.56)	0.003
Regular exercise				
Yes	237	1.15 (0.95, 1.38)	1.33 (0.89, 1.99)	0.159
No	1122	1.14 (1.04, 1.25)	1.32 (1.10, 1.60)	0.004
BMI				
BMI < 25, normal	1153	1.15 (1.05, 1.26)	1.34 (1.10, 1.62)	0.003
BMI ≥ 25, overweight	206	1.13 (0.94, 1.37)	1.30 (0.87, 1.93)	0.198

Adjusted for age, gender, current smoker, current drinker, regular exercise, BMI and CVDs (hypertension, heart disease, atrial fibrillation and diabetes).

MDA, malondialdehyde; BMI, body mass index; CVDs, cardiovascular diseases.

**Table 5:** Stratified analysis for relationship between blood MDA level and stroke risk in participants from City&town. (N = 239).

Variables	N	OR, 95%CI	PerSD change	P value
Age				
Age < 84	133	0.79 (0.43–1.44)	0.66 (0.23–1.89)	0.438
Age ≥ 84	106	0.98 (0.60–1.59)	0.96 (0.41–2.24)	0.927
Gender				
Male	117	0.92 (0.60–1.42)	0.87 (0.41–1.84)	0.716
Female	122	1.05 (0.63–1.73)	1.08 (0.45–2.60)	0.855
Current smoker				
Yes	37	–	–	–
No	202	0.96 (0.68–1.34)	0.93 (0.51–1.67)	0.798
Current drinker				
Yes	29	–	–	–
No	210	0.94 (0.67–1.31)	0.89 (0.50–1.59)	0.700
Regular exercise				
Yes	45	–	–	–
No	194	0.84 (0.58–1.24)	0.75 (0.38–1.45)	0.387
BMI				
BMI < 25, normal	202	0.79 (0.49–1.26)	0.66 (0.29–1.50)	0.325
BMI ≥ 25, overweight	37	0.75 (0.22–2.58)	0.60 (0.07–5.20)	0.644

Adjusted for age, gender, current smoker, current drinker, regular exercise, BMI and CVDs (hypertension, heart disease, atrial fibrillation and diabetes).

MDA, malondialdehyde; BMI, body mass index; CVDs, cardiovascular diseases.

## Discussion

In this cross-sectional and community-based study, after adjustment for important identified confounders, blood MDA levels presented a positive association with stroke risk among adults in the Chinese elderly population. Specifically, higher values of serum MDA predicted an elevated risk of stroke. After we explored this association in the urban‒rural stratification analysis of these participants, the results further supported an urban‒rural difference in the association. Elevated blood MDA levels were independently associated with increased stroke risk only in rural individuals but not in urban individuals.

Oxidative damage caused by ROS can result in cell death and tissue destruction, such as protein denaturation, lipid peroxidation, DNA damage, inactivation of enzymes, release of Ca^2+^ from intracellular stores, and destruction of the cytoskeletal structure. The condition of ischemia and reperfusion after stroke has been found to be associated with overproduction of ROS, potentially leading to neuronal death^24–28^. MDA is commonly used as a marker of oxidative status and is related to the excessive formation of ROS^25^. Considering the adverse effect of oxidative stress on stroke, we analyzed the relationship between serum MDA and the risk of stroke. As we expected, our results showed that serum MDA levels were significantly and positively associated with the risk of stroke, which is consistent with previous conclusions that excessive oxidative damage increases the risk of stroke^25,28^. Interestingly, we found an urban‒rural difference in the association that elevated blood MDA levels were independently associated with an increased risk of stroke in rural individuals but not in urban individuals. Some previous evidence may partly explain this discrepancy. For instance, there is evidence to report that rural individuals are likely to have worse risk factor profiles of stroke, such as unhealthy lifestyles (high mental pressure, staying up late to work and less exercise) and eating habits (high fat and high sugar diet), related to higher levels of oxidative stress, which might contribute to a higher stroke risk, compared with urban individuals^1–4^. Because of improved living standards in China, a high-fat diet and unhealthy lifestyle associated with oxidative stress and its related diseases are more common in rural populations. It is also known that individuals from rural areas tend to have more risk factors for stroke, including hypertension, diabetes and dyslipidemia, than those from urban areas^5–9^. In addition, early prevention and treatment of stroke are not well performed in rural populations, which leads to a high risk of apoplectic attack due to a lack of health awareness. These reasons may partly explain why elevated MDA levels can be associated with higher stroke risk in rural participants than in urban participants. Certainly, if we would like to elucidate this urban‒rural difference from the perspective of molecular mechanisms, it is still quite difficult due to numerous complex mechanisms concerning stroke and its related risk factors, and further research may be needed in the future.

Additionally, to analyze the impact of other variables, such as sociodemographic characteristics and lifestyle, stratification analyses were used to further evaluate the independent association between blood MDA levels and stroke risk. Importantly, we observed that age, current drinking status, regular exercise and BMI in rural participants had significant influences on the association of blood MDA with stroke risk. The results further support our view that excessive production of MDA caused by oxidative stress in rural populations contributed to the higher stroke risk. However, the effect of these factors in the urban participants was not significant, which may be because the urban population is likely to have better lifestyle habits, a healthier diet and more medical resources, leading to a relatively lower incidence rate of stroke.

In this cross-sectional study, we first found an independent association between increased blood MDA levels and the risk of stroke among old adults. Specifically, the independent association only existed in rural individuals but not in urban individuals, which extends few previous studies on urban‒rural differences related to stroke in the elderly population. However, several limitations should be noted in the present study. First, blood indicators were tested only once. In the process of stroke, different levels of oxidative stress lead to different levels of blood MDA, which may further make our analysis biased. Second, more than 9000 individuals were included in the CLHLS study. Only 2439 individuals participated in blood biochemical tests, and 1598 individuals were included in our study because approximately 800 individuals were excluded due to the absence of important variables. Our study did not significantly analyze data on these excluded populations. Finally, we cannot define the type of stroke (hemorrhagic or ischemic stroke, acute or chronic stroke), which might produce a heterogeneous association between serum MDA and the risk of different stroke types.

## Conclusions

Our results indicated that elevated blood MDA levels were related to increased stroke risk in rural individuals but not in urban individuals. This may provide new information that excessive oxidative stress contributes to increased stroke risk in rural populations.

## Data Availability

Study data were from the CLHLS study (https://www.icpsr.umich.edu/web/pages/ICPSR/), which is a prospective, longitudinal, community-based study. Twenty-two provinces in China were initially selected, and then half of the cities or counties in these provinces were randomly selected to include the study population. A detailed description of the CLHLS has also been published elsewhere^20^. The datasets used and/or analyzed during the current study are available from the corresponding author upon reasonable request.
